# Gun-Shot Fractures of the Extremities

**Published:** 1919-01

**Authors:** 


### GUN-SHOT FRACTURES OF THE EXTREMITIES

In his treatise on surgery, the elder Gross expressed a sentiment that many surgeons have felt during the war that has just ended, when he wrote : "It is always with fear and trembling that I undertake the treatment of even the simplest fracture, because no man can foretell the result ". Gross lived before the days of the X-ray, asepsis was in its infancy when he died, and there have been many advances in the mechanical treatment of fractures since he practiced surgery; but with all the improvements in surgical technic in the past half century, there have been many disappointments to surgeons in the treatment of gun-shot fractures of the extremities that have been so frequent among our wounded soldiers. There is no question, however, that, generally speaking, the results which have been obtained in the treatment of fractures in the recent war have been far better than ever before.

The good results that have been attained in treating fractures have been in direct proportion to the aseptic technic of surgeons and to their skill and experience in applying the mechanical devices that have been perfected, or that have been improved upon in the past four years. The surgeons who failed to familiarize themselves with the application of the

Thomas and other splints for the immobilization of fractured limbs, and those who have not developed the "aseptic technic", are the ones who have had the most failures. If the time that was spent in Medical Officers' Training Camps in trying to make military men out of civilian doctors had been utilized in teaching them how to apply splints, and how to treat wounds, the end results in the treatment of gun-shot fractures would have been much better, because the average physician in the United States had had but little experience in treating fractures of this kind prior to the war, and he had had almost no opportunity for acquiring a good technic in the treatment of gun-shot injuries to bones and joints.

One of the first of the great surgeons of the United States who came to France was Colonel Joseph A. Blake, who, in 1914, was placed in charge of the American Ambulance Hospital in Paris. He perhaps has had a larger experience in the treatment of gun-shot fractures of the extremities than any other American in France. Throughout the four years in which his work has been entirely in war surgery, most of it of the extremities, he has treated many thousands of cases of gun-shot fractures. He and his confreres in the American Ambulance Hospital, which soon after the entry of the United States into the war was transferred to the American Army and called American Military Hospital No. 2, have simplified and improved the methods for the treatment of compound fractures that have been in use for many years. They have also developed some new methods of treatment.

Colonel Blake, in the book called " Gun-shot Wounds of the Extremities ", has recorded his conclusions, and he has described the methods of treatment of war wounds of bones and joints used in the hospital of which he is Commanding Officer. In it he discusses the mechanism and varieties of gun-shot fractures and their repair. The transportation and operative treatment of fractures, in general, is also discussed, but the major part of the work is given to descriptions, with many excellent cuts, of the methods of mechanical treatment with the apparatus which he has found useful in his work. The chapter on wounds of the joints is also a very important one. The book is of particular value because the author has described only those methods which he has tried and knows are satisfactory.

Believing that every medical officer in the army should have the benefit of this latest and perhaps best treatise on "Gun-shot Wounds of the Extremities", the American Red Cross has purchased a sufficient number of copies to provide one for each medical officer in the American Expeditionary Forces. There have been some difficulties connected with the distribution of literature sent by the American Red Cross to medical officers of the American Expeditionary Forces, and if any officer fails to receive a copy of this manual on fractures, he may have one sent him by writing, or applying, to the American Red Cross Medical Library, 12 Place Vendome, Paris.

